# Integrating spatial omics with routine haematoxylin and eosin in formalin-fixed paraffin-embedded: a step-by-step clinical workflow

**DOI:** 10.12688/f1000research.170680.1

**Published:** 2025-10-09

**Authors:** Nasar Alwahaibi

**Affiliations:** 1 Biomedical Science, Sultan Qaboos University College of Medicine and Health Science, Muscat, Muscat Governorate, Oman

**Keywords:** Spatial omics; FFPE, histopathology, H&E, in situ RNA imaging, imaging mass cytometry, multiplex ion beam imaging.

## Abstract

Haematoxylin and eosin (H&E) remain the foundation of tissue diagnosis, yet many clinical questions, tumour–immune architecture, spatial heterogeneity, and predictors of therapy response, require molecular context that routine slides cannot provide. Spatial omics closes this gap by mapping RNA and proteins in situ while preserving morphology, and recent platforms are increasingly compatible with formalin-fixed paraffin-embedded (FFPE) tissue, enabling use in routine pathology and retrospective cohorts. This mini-review offers a pragmatic, step-by-step workflow for integrating spatial assays with H&E: define the clinical decision; select a fit-for-purpose modality (whole-transcriptome spot/grid vs targeted in situ RNA; multiplex proteomics); lock pre-analytics aligned to histology (sectioning, staining, de-crosslinking, storage); pre-specify regions of interest (ROIs), registration, and segmentation rules; analyse with quality-assurance gates (normalisation, deconvolution, batch handling, spatial statistics); and validate and report using orthogonal assays and multi-site replication. FFPE-ready platforms and typical use-cases are summarised, with emphasis on pre-analytical factors that materially affect signal and analysis “recipes” distilled from recent benchmarks. Brief clinical exemplars illustrate how H&E-anchored spatial maps change decisions by pinpointing actionable niches (e.g., immune neighbourhoods, vascular niches, layer-specific programmes). Common limitations are also outlined, including technology trade-offs, pre-analytics, sampling bias, segmentation and deconvolution error, batch effects, cost, turnaround, and regulatory considerations. Future directions include standards and metadata, cross-platform integration, prospective evidence, automation and quality assurance, and multi-omic detection. Overall, the goal is to support pathology and translational teams in adopting spatial omics in FFPE with both discipline and speed, focusing on clinically meaningful decisions while ensuring reproducibility and credibility.

## Introduction

Histopathology still begins with haematoxylin and eosin (H&E), yet many clinical questions about tumour–immune architecture, heterogeneity, and therapy response, require molecular context that routine slides cannot provide. Spatial omics helps close this gap by mapping RNA and proteins in situ while preserving tissue architecture, and in the past few years platforms have become increasingly formalin-fixed paraffin-embedded (FFPE) compatible, widening access for routine pathology and retrospective biobanks. ^1, 2^ High-resolution spatial transcriptomics can localise billions of transcripts at subcellular scales, supporting detailed maps of cell–cell interactions in clinical material and opening avenues for research and patient care. ^3, 4^ Recent overviews aimed at pathologists and translational teams underscore this momentum and its implications for clinical research. ^5– 7^

Despite rapid progress, barriers to confident adoption persist. Common pain points include: pre-analytical variability (fixation, sectioning, de-crosslinking), unclear best practices for region-of-interest (ROI) selection and cell segmentation, analytical and batch effects across slides/cohorts, and uncertainty about validation and reporting standards that will satisfy clinical rigour. Methodological reviews and best-practice guide repeatedly call out these gaps, and highlight the need for clearer guidance on how to integrate spatial readouts with H&E across the biopsy-to-report workflow. ^8– 10^

This mini-review responds to those needs with a practical, FFPE-focused roadmap for pathology services and translational laboratories. It compares widely used FFPE-ready platforms, sequencing-based spatial transcriptomics (e.g., Visium/Visium HD) ^11^ and imaging-based in situ platforms (e.g., Xenium, CosMx), ^12^ in terms of resolution, panel breadth, capture area, and typical use-cases, ^13^ distills pre-analytics and QC steps aligned to histology workflows, ^14^ outlines ROI design, registration, segmentation, and analysis “recipes” that survive peer review, ^15^ and summarises validation strategies, including orthogonal assays and multi-site replication. ^16– 18^ By anchoring recommendations in platform documentation and recent translational reviews, the focus remains on choices that are feasible in FFPE and compatible with routine pathology. ^19^

**Figure 1.**
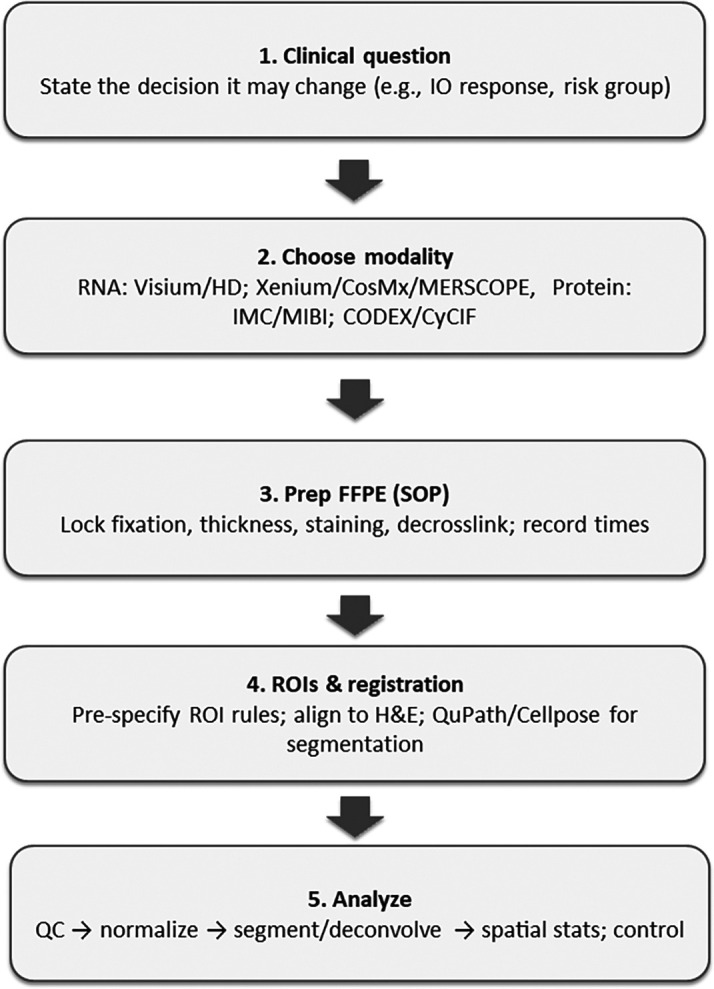
Spatial and haematoxylin and eosin analysis in formalin-fixed paraffin-embedded: streamlined vertical workflow from decision-making to reporting.

The aim of this mini-review is to provide a step-by-step guide for integrating spatial omics with routine H&E in FFPE specimens so teams can select a fit-for-purpose modality, implement robust pre-analytics and QC, plan analyses that generalize across sites, and structure validation and reporting to accelerate translational impact. Figure 1 summarises the end-to-end FFPE spatial workflow, define the decision → select modality → lock pre-analytics → pre-specify ROIs & registration → analyse with QA gates → validate & report, which I use to organise the sections that follow.

## Platforms for FFPE pathology: what actually works

Spatial assays you can deploy on archival FFPE tissue fall into two broad camps. Sequencing-based spatial transcriptomics (ST), e.g., 10x Visium HD (FFPE), captures spot-based whole-transcriptome profiles registered to H&E, trading single-cell resolution for large capture areas and broad gene coverage. ^20^ In situ imaging platforms, e.g., 10x Xenium and NanoString CosMx SMI, measure targeted RNA (and, for CosMx, proteins) at single-cell or subcellular resolution on FFPE sections. ^21, 22^ MERFISH/MERSCOPE (Vizgen) is another high-plex in situ option with FFPE support. ^23^ For multiplex spatial proteomics, laboratories commonly use Imaging Mass Cytometry (IMC), Multiplexed Ion Beam Imaging (MIBI), ^24, 25^ or cyclic immunofluorescence systems such as CODEX/CyCIF ^26^; these often align naturally with IHC-centric diagnostic questions. Good platform overviews for pathologists are now available, alongside manufacturer FFPE handbooks. Key specifications are summarised in Table 1: sequencing-based “spot/grid” assays (e.g., 10x Visium FFPE/Visium HD) provide whole-transcriptome discovery over 6.5 × 6.5 mm capture areas (Visium 55 μm spots; HD 2 μm pixel output, typically binned), well suited to archival cohort screens and tumour–stroma mapping. ^27, 28^ In situ RNA imaging (10x Xenium, NanoString CosMx SMI, Vizgen MERSCOPE) yields targeted single-cell/subcellular maps (CosMx up to ~6,000 RNAs; MERSCOPE up to ~1,000) for pathway-focused profiling, immune-niche interrogation, and cross-validation with IHC/RNAscope. ^29– 31^ Multiplex spatial proteomics (IMC, MIBI, CODEX, CyCIF) complements RNA by quantifying proteins at single-cell resolution for immune phenotyping and actionable signatures. ^32^

**Table 1.** Formalin-fixed paraffin-embedded -compatible spatial omics platforms: a concise comparison for routine pathology.

Class	Representative platforms	Nominal resolution	Analyte	Panel breadth	Capture area/FOV	Typical throughput	Typical use-cases	References
Sequencing-based ST (spot/grid)	10x Visium FFPE/Visium HD	Visium: 55 μm spots; HD: 2 μm pixel output (binned for analysis)	RNA (whole-transcriptome)	Whole-transcriptome	Up to 4 capture areas/slide (~6.5 × 6.5 mm each)	Tens of sections per run (scanner + NGS dependent)	Discovery in archival cohorts; tumor–stroma programs; hypothesis generation	^27, 28^
In-situ RNA imaging	10x Xenium; NanoString CosMx SMI; Vizgen MERSCOPE	Single-cell/subcellular	RNA (targeted); CosMx also protein	Hundreds–thousands RNAs (CosMx up to ~6,000; MERSCOPE up to ~1,000); CosMx ~64–76 proteins	Tile-based FOVs; user-selected ROIs; multi-tile mosaics	~1–10 slides/week (instrument dependent)	Targeted pathway panels; immune niches; cross-validation with IHC/RNAscope	^29– 31^
Multiplex spatial proteomics	IMC; MIBI; CODEX; CyCIF	Single-cell	Protein (antibody panels)	~30–60+ markers (panelized)	Tile ROIs; mm ^2^–cm ^2^ mosaics	~1–10 slides/week	Immune phenotyping; actionable protein signatures; trial correlative studies	^32^

ST, spatial transcriptomics; FFPE, formalin-fixed paraffin-embedded; HD, high-definition pixel output (Visium HD); NGS, next-generation sequencing; RNA, ribonucleic acid; FOV, field of view; ROI, region of interest; IHC, immunohistochemistry; RNAscope, RNA in situ hybridization; SMI, Spatial Molecular Imager; IMC, imaging mass cytometry; MIBI, multiplexed ion beam imaging; CODEX, CO-Detection by indEXing; CyCIF, cyclic immunofluorescence; μm, micrometres; mm ^2^, square millimetres; cm ^2^, square centimetres.

## Pre-analytics & tissue handling: small decisions, big effects

FFPE spatial assays are unusually sensitive to pre-analytics. ^33^ Follow platform-specific guidance on section thickness, deparaffinisation, H&E/IF staining, decrosslinking, and storage; these steps strongly influence RNA integrity, probe binding, and downstream quantification. ^34^ For example, the Visium HD FFPE handbook and Xenium FFPE guide detail slide prep, staining, and decrosslinking workflows ^27^; MERSCOPE provides FFPE-specific drying and storage advice. Critically, enzymatic steps can backfire: excess Proteinase-K in GeoMx DSP improved total reads but increased negative probe counts and reduced signal-to-noise, ultimately decreasing genes detected, highlighting why labs should pilot enzyme conditions and lock them before a study. ^31^

## ROI selection, registration & segmentation

ROI strategy should be hypothesis-driven (e.g., tumour–stroma interfaces, immune niches, invasive fronts) and traceable back to H&E. ^35^ Platforms such as GeoMx and in situ imagers emphasize explicit ROI selection; document criteria prospectively. ^36^ Register spatial layers to H&E and use validated, reproducible segmentation—QuPath remains a robust open-source WSI toolset for nuclei/cell detection, while Cellpose (and its newer variants) generalizes well across staining modalities with minimal tuning. ^37^ When publishing multiplex imaging data, adhere to the Minimum Information about Highly Multiplexed Tissue Imaging (MITI) standard so ROIs, acquisition parameters, and processing are transparent and reusable. ^38, 39^

## Analysis workflows that survive peer review

For spot-based spatial transcriptomics, most groups (a) perform QC and normalisation, ^40, 41^ (b) deconvolve spots with scRNA-seq references, ^42, 43^ and (c) test spatial associations. ^44^ Recent benchmarking across dozens of datasets recommends cell2location, CARD, and Tangram as consistently high performers ^45^; newer methods continue to appear, but your review should point readers to benchmark-grounded choices. For multi-slice or multi-cohort integration, use modern alignment tools and report cross-slide consistency. ^46^ For imaging proteomics, denoising, batch correction, and neighbourhood analysis are critical ^47^; recent best-practice pieces in oncology outline end-to-end pipelines (acquisition → segmentation → phenotyping → spatial stats) ^9, 45, 48, 49^

## Validation & reproducibility

Translational claims require orthogonal validation (e.g., RNAscope/IHC for RNA/protein hits), multi-site replication, and pre-registered analysis plans. ^50^ Use reporting checklists from pathology-facing reviews and adopt MITI for multiplex imaging so images, masks, and metadata are reusable. ^37^ Where possible, include an external test set (a different scanner/site or archival cohort) and quantify agreement (e.g., correlation of cell-type abundance, niche frequency). ^51^ High-level clinical perspectives emphasize linking spatial findings to outcomes or therapeutic response, not just discovery. ^52^

## Costs, throughput, and choosing RNA vs protein maps

For budgeting and platform choice, compare assay chemistry, resolution, capture area/fields per run, and instrument time rather than chasing absolute prices (which vary by site and service contract). As a guide, instrument cost can be considered high (>$500,000), medium ($100,000–$500,000), or low (<$100,000); per-sample cost high (>$1,000), medium ($100–$1,000), or low (<$100). ^53^ Protein-centric maps (multiplex IHC/IF) often deliver faster, lower per-slide costs for focused questions (e.g., immune phenotyping), ^54^ whereas whole-transcriptome ST (UMI-based RNA profiling) is better for unbiased discovery and retrospective cohorts. Resolution needs, spot vs single-cell/subcellular, and capture area (including the effective pixel/“bin,” e.g., 100 μm ^2^) determine run time and sequencing/imaging depth. ^55^ Above all, FFPE compatibility and workflow fit (embedding within existing histology/QC) should drive selection; LCM remains useful for targeted validation or rare regions. ^56^ Manufacturer documents (e.g., 10x Genomics, NanoString, Vizgen) summarize throughput, section prep, and run constraints that materially affect real-world cost and turnaround. ^12, 57, 58^

## Adoption roadmap for pathology services

Start small. Define a narrow clinical question and the decision it might change; pick one FFPE-compatible platform and standardize pre-analytics; write down ROI rules and lock segmentation; pre-register analysis and plan orthogonal validation; follow MITI for data/metadata; include a multi-site or external test component as early as feasible. ^59, 60^ Recent best-practice frameworks in multiplex imaging/spatial biology, plus pathology-specific reviews, provide checklists you can adapt to your SOPs and QA documents. ^24^

## Clinical exemplars

Below are brief, real-world examples showing how pairing spatial omics with routine H&E can change decisions. By revealing what is happening and exactly where in the tissue, these maps help clinicians choose the right biopsy area, refine risk, and pick or validate targets for therapy, things that routine H&E or bulk tests often miss.

In cutaneous squamous cell carcinoma, pairing spatial omics with H&E revealed where distinct tumour programmes live and whom they talk to. Integrated single-cell RNA-seq, spatial transcriptomics, and multiplexed ion-beam imaging mapped four tumour subpopulations, including a tumour-specific keratinocyte (TSK) state that localises to a fibrovascular niche on the H&E slide. Spatial mapping of ligand–receptor networks showed TSK cells act as a communication hub, while Tregs co-localized with CD8 T cells in compartmentalized stroma, an immunosuppressive arrangement you could miss with bulk profiling. Functionally, CRISPR screens flagged subpopulation-enriched networks as essential for tumourigenesis. Clinically, these H&E-anchored spatial readouts can guide biopsy targeting (sample the TSK/fibrovascular interface), refine risk stratification (presence/extent of Treg–CD8 niches), and nominate actionable pathways for trials focused on interrupting TSK-driven signalling or collapsing immunosuppressive neighbourhoods. ^61^

In pancreatic ductal adenocarcinoma, overlaying spatial proteomics on the H&E slide mapped the tumour microenvironment into 10 distinct neighbourhoods, including a vascular niche within PDAC’s characteristically hypovascular, hypoxic stroma. Across 35 H&E-guided ROIs from 9 patients (>140k cells, 26-marker imaging mass cytometry), the study localized where tumour proliferation concentrates and how immune subsets interface with vessels. Crucially, the vascular niche was tightly linked to CD44 ^+^ macrophages with a pro-angiogenic programme, nominating a microenvironmental target that standard bulk assays would miss. Clinically, these H&E-anchored spatial readouts can guide biopsy targeting (sample vascular niches), sharpen risk stratification (proliferative/immune–vascular interfaces), and inform trial design for anti-angiogenic or macrophage-modulating combinations in PDAC. ^62^

In fatal COVID-19 lung disease, FFPE spatial transcriptomics (GeoMx) co-registered to H&E pinpointed patchy, non-uniform SARS-CoV-2 distribution and localised host responses to the exact anatomic foci. Areas with high viral load on the slide showed amplified type I interferon signaling, alongside broader upregulation of inflammation, coagulation, and angiogenesis pathways, patterns a bulk assay would blur. After controlling for dominant cell types and inter-patient variability, only a few genes distinguished COVID-19 from fatal influenza, but IFI27 remained significantly higher in COVID-19, reinforcing its value as a tissue-level biomarker that aligns with blood-based diagnostics. Clinically, H&E-anchored spatial readouts can guide targeted sampling (multiple foci rather than single cores), support triage/therapy decisions by confirming interferon-rich, highly infected regions, and validate biomarkers like IFI27 directly in diseased lung architecture. ^63^

In human dorsolateral prefrontal cortex, H&E-anchored spatial transcriptomics (10x Visium) mapped the six cortical layers and uncovered layer-enriched gene programmes, refining classic laminar markers on the same slide. Overlaying these maps onto single-nucleus RNA-seq re-grounded molecular clusters in real anatomy, improving interpretability. Clinically relevant gene sets for schizophrenia and autism showed layer-specific enrichment, pointing to circuits and cell layers most implicated in disease, insight that can guide targeted sampling, neuropathology reporting, and hypothesis-driven trials (e.g., layer-aware biomarkers or neuromodulation targets). A simple data-driven clustering workflow further supports tissues with less obvious architecture, using H&E context to define spatial domains when boundaries are not visually clear. ^64^

In periodontitis, H&E-anchored spatial transcriptomics resolved gingival tissue into epithelium, inflamed connective tissue, and non-inflamed connective tissue on the same slide, revealing 92 genes upregulated specifically in inflamed zones. Top signals, IGLL5, SSR4, MZB1, XBP1, point to a B-cell/plasma-cell–rich, high-secretory programme and were validated by RT-qPCR and IHC. Clinically, these maps let dentists and pathologists target biopsies to truly active lesions, distinguish active vs quiescent sites for risk stratification and follow-up, and track response to therapy using compartment-specific markers, insights that bulk profiling would average away. ^65^

In melanoma lymph node metastases, H&E-anchored spatial transcriptomics (10x Visium) sequenced >2,200 tissue domains and, after deconvolution, linked gene programmes to specific histological entities on the slide. This revealed coexisting melanoma transcriptional signatures within single regions and defined lymphoid niches adjacent to tumour with distinct expression patterns, heterogeneity not evident on morphology alone. Clinically, such maps can refine biopsy targeting (sample mixed-signature zones), sharpen staging/prognosis by quantifying tumour–immune interfaces, and inform immunotherapy strategies by identifying lymphoid areas most engaged with tumour. In short, pairing spatial omics with H&E exposes actionable intratumoural and microenvironmental complexity that bulk profiling and routine histology would miss. ^66^

In rheumatoid arthritis (RA) vs spondyloarthritis (SpA) synovium, H&E-anchored spatial transcriptomics let investigators zoom into mononuclear infiltrates on the slide and read out compartment-specific programmes. RA hotspots showed adaptive immune/T–B cell interaction signatures with enrichment of central memory T cells, whereas SpA regions favoured tissue-repair pathways with effector memory T cells. These H&E-guided spatial maps, validated by IHC and in silico cell-type calls, offer practical levers: refine biopsy targeting, support differential diagnosis when histology overlaps, and align therapy choices (e.g., B/T-cell–directed strategies in RA vs repair-oriented pathways in SpA) while enabling site-specific response monitoring. ^67^

In leprosy, pairing spatial omics with H&E turned granulomas from a uniform “mass” on the slide into an organised, layered architecture with distinct cellular and functional zones. By integrating single-cell and spatial sequencing on biopsies from reversal reactions (RRs) versus lepromatous disease (L-lep), the study localised interferon-γ/IL-1β–regulated antimicrobial programmes to specific niches where macrophages, T cells, keratinocytes, and fibroblasts cooperate. Clinically, H&E-anchored maps can guide targeted sampling of active antimicrobial layers during RR, inform biomarker development for treatment monitoring (spatially resolved antimicrobial gene sets), and support therapy tailoring by highlighting sites most likely to respond to host-directed or immunomodulatory interventions, granularity that bulk assays or morphology alone would miss. ^68^

In ALS cortex, H&E-anchored spatial transcriptomics (∼100 μm spots) preserved laminar and regional anatomy on the slide, letting investigators pinpoint where disease programmes reside rather than averaging them out. Mapping post-mortem motor cortex from a C9orf72 case, then validating with BaseScope ISH and an extended cohort (sALS, SOD1, C9orf72), they found 16 dysregulated transcripts spanning six disease pathways and converged on two spatially dysregulated genes, GRM3 and USP47, consistently altered across ALS genotypes. Clinically, these H&E-registered maps help explain selective regional vulnerability, nominate region-aware diagnostic markers and therapeutic targets, and guide targeted sampling in neuropathology, insights that bulk RNA or dissociated single-cell data would miss. ^69^

In another ALS, H&E-anchored spatial transcriptomics mapped the spinal cord’s molecular shifts across disease time in mice and in human post-mortem tissue, revealing when and where key pathways turn on. The maps distinguished regional microglia vs astrocyte programmes early in disease, and identified transcriptional pathways shared between murine models and human cords, signals that bulk RNA or dissociated cells would blur. Clinically, these slide-localized readouts can guide targeted sampling (vulnerable ventral horn regions), sharpen biomarker development (region- and cell-state markers for progression), and inform trial design/stratification (enrolling patients by pathway-active niches), aligning therapeutic timing with the actual spatial order of neuroinflammatory events. ^70^

## Limitations of spatial omics

Limitations of spatial omics span several practical areas. First, there are technology trade-offs on FFPE sections between map resolution, number of targets, and area covered ^71^: whole-transcriptome spot/grid methods lose single-cell precision, targeted in situ platforms measure fewer genes, and multiplex proteomics depends on well-validated antibodies. ^72^ Second, results are sensitive to pre-analytics, fixation quality, section thickness, and deparaffinisation/de-crosslinking, where too little or too much enzyme treatment degrades data; even storage time of cut slides matters. ^73– 75^ Certain tissues are difficult: decalcified bone often has fragmented RNA, necrotic/bleeding areas give weak signal, and highly pigmented/autofluorescent tissues (e.g., melanoma, lipofuscin-rich) can confound fluorescence without mitigation. ^64, 76^ Third, study design and sampling can introduce bias if ROI rules are not pre-specified and auditable (MITI) ^49, 77^; small ROIs may be underpowered and single-slide studies face slide/batch variability, so plan power, use multiple slides, and model batch. ^78– 80^ Retrospective cohorts may hide clinical/treatment confounders, so follow Strengthening the Reporting of Observational Studies in Epidemiology (STROBE)/Reporting Recommendations for Tumor Marker Prognostic Studies (REMARK) principles. ^81, 82^ Fourth, quantification and analysis have pitfalls: segmentation/cell calling remains error-prone and software/version changes can shift results, pipelines and parameters should be locked. ^83, 84^ For spot-based data, deconvolution depends on single-cell references that may not match tissue/platform/disease and can bias estimates ^85– 87^; batch effects (slide/run/site) can masquerade as biology without careful normalization/integration ^88^; testing thousands of features inflates false positives unless False Discovery Rate (FDR) is controlled and primary hypotheses are pre-registered. ^89^ Fifth, validation and generalizability are limited by variable cross-platform concordance (sequencing- vs imaging-based), so orthogonal confirmation (RNAscope/IHC) is important ^64, 90^; many studies stop at discovery rather than prospective, multi-site validation with outcomes and REMARK-aligned reporting. ^18, 91^ Sixth, operational and regulatory barriers include cost, compute/storage, and turnaround (e.g., Visium FFPE/HD depth; multi-TB images and QC), ^49, 92^ site-to-site differences in infrastructure/training/QA that hinder reproducibility, ^93^ and the need to move from Research Use Only/Laboratory-Developed Test (RUO/LDT) RUO/LDT to in vitro Diagnostic (IVD) through Clinical Laboratory Improvement Amendments/College of American Pathologists (CLIA/CAP)-level validation with ongoing monitoring for assay/model drift. ^94^

## Future directions

To bring spatial omics into routine pathology, we need simple shared rules for data collection and reporting, using MITI-style metadata/checklists and multi-site harmonization, so ROI choices are auditable and datasets can be compared across studies. ^4, 64^ We also need better ways to integrate platforms: align whole-transcriptome maps with targeted in situ RNA and multiplex proteomics on serial sections, and quantify uncertainty in those integrations, building on recent cross-technology benchmarks. ^91, 95, 96^ Clinical adoption will require prospective, multi-site studies with predefined endpoints, external test cohorts, and reporting aligned to biomarker standards such as REMARK. ^97^ End-to-end automation and QA, registration, segmentation/cell calling, deconvolution, batch correction, with version-locked code and continuous QC dashboards should be standard. ^40, 98^ Practical multi-omic co-detection protocols (RNA–protein now, metabolites later) on FFPE, paired with orthogonal validation (RNAscope/IHC), will increase confidence. ^83, 99^ Finally, improving cost and throughput, through batching, smart ROI strategies, and targeted panels, will help meet clinical turnaround times; recent work outlines feasible high-throughput paths. ^37, 39^

## Conclusions

Spatial omics now complements routine H&E on FFPE tissue and can answer clinically relevant questions about tumour–immune architecture, heterogeneity, and microenvironmental niches. Effective adoption hinges on four elements emphasized in this mini-review: (1) fit-for-purpose platform selection (RNA vs protein; discovery vs targeted), (2) disciplined pre-analytics and QC, (3) transparent ROI, registration, and analysis workflows that are locked and auditable, and (4) orthogonal validation and multi-site replication to support translational claims. Framing studies around decisions that matter to clinicians (diagnosis, risk stratification, therapy selection) will accelerate real-world impact.

## Data Availability

There are no underlying data associated with this article.
