# Integrating spatial omics with routine haematoxylin and eosin in formalin-fixed paraffin-embedded: a step-by-step clinical workflow

**DOI:** 10.12688/f1000research.170680.2

**Published:** 2026-01-19

**Authors:** Nasar Alwahaibi

**Affiliations:** 1 Biomedical Science, Sultan Qaboos University College of Medicine and Health Science, Muscat, Muscat Governorate, Oman

**Keywords:** Spatial omics; FFPE, histopathology, H&E, in situ RNA imaging, imaging mass cytometry, multiplex ion beam imaging.

## Abstract

Haematoxylin and eosin (H&E) remain the foundation of tissue diagnosis, yet many clinical questions, tumour–immune architecture, spatial heterogeneity, and predictors of therapy response, require molecular context that routine slides cannot provide. Spatial omics closes this gap by mapping RNA and proteins in situ while preserving morphology, and recent platforms are increasingly compatible with formalin-fixed paraffin-embedded (FFPE) tissue, paving the way for its future potential use in routine pathology and retrospective cohorts. However, significant challenges related to cost, complexity, reproducibility, and regulation currently remain before widespread routine deployment, underscoring that it is not yet ready for In Vitro Diagnostic or routine clinical application. This mini-review offers a pragmatic, step-by-step workflow for integrating spatial assays with H&E: define the clinical decision; select a fit-for-purpose modality (whole-transcriptome spot/grid vs targeted in situ RNA; multiplex proteomics); lock pre-analytics aligned to histology (sectioning, staining, de-crosslinking, storage); pre-specify regions of interest (ROIs), registration, and segmentation rules; analyse with quality-assurance gates (normalisation, deconvolution, batch handling, spatial statistics); and validate and report using orthogonal assays and multi-site replication. FFPE-ready platforms and typical use-cases are summarised, with emphasis on pre-analytical factors that materially affect signal and analysis “recipes” distilled from recent benchmarks. Brief clinical exemplars illustrate how H&E-anchored spatial maps change decisions by pinpointing actionable niches (e.g., immune neighbourhoods, vascular niches, layer-specific programmes). Common limitations are also outlined, including technology trade-offs, pre-analytics, sampling bias, segmentation and deconvolution error, batch effects, cost, turnaround, and regulatory considerations. Future directions include standards and metadata, cross-platform integration, prospective evidence, automation and quality assurance, and multi-omic detection. Overall, the goal is to support pathology and translational teams in adopting spatial omics in FFPE with both discipline and rigor, guiding the necessary steps to ensure reproducibility and credibility for eventual clinical impact.

## Introduction

Histopathology still begins with haematoxylin and eosin (H&E) and relies heavily on immunohistochemistry (IHC) for clinical decision-making. However, many clinical questions about tumour–immune architecture, heterogeneity, and therapy response, require a depth of molecular context and objective quantification that even high-plex H&E and IHC alone cannot fully provide. Spatial omics helps close this gap by mapping RNA and proteins in situ while preserving tissue architecture, uniquely offering high-plex co-localization, precise delineation of cellular niches and molecular gradients, detailed architectural context, and quantitative data. In the past few years, platforms have become increasingly formalin-fixed paraffin-embedded (FFPE) compatible, widening access and opening avenues for high-quality translational research and eventual integration into routine pathology and retrospective biobanks. ^1, 2^ It is critical to note that despite this potential, spatial omics is not currently poised for In Vitro Diagnostic (IVD) or routine clinical application due to inherent complexities and validation needs. High-resolution spatial transcriptomics can localise billions of transcripts at subcellular scales, supporting detailed maps of cell–cell interactions in clinical material and opening avenues for research and patient care. ^3, 4^ Recent overviews aimed at pathologists and translational teams underscore this momentum and its implications for clinical research. ^5– 7^

Despite rapid progress, barriers to confident adoption persist. Recurring challenges include: pre-analytical variability (fixation, sectioning, de-crosslinking), the lack of established best practices for region-of-interest (ROI) selection and cell segmentation, analytical and batch effects across slides/cohorts, and uncertainty about validation and reporting standards that will satisfy clinical rigour. Methodological reviews and best-practice guide repeatedly call out these gaps, and highlight the need for clearer guidance on how to integrate spatial readouts with H&E across the biopsy-to-report workflow. ^8– 10^

This mini-review responds to those needs with a practical, FFPE-focused roadmap for research and translational laboratories, outlining a disciplined path forward for eventual clinical implementation. It compares widely used FFPE-ready platforms, sequencing-based spatial transcriptomics (e.g., Visium/Visium HD) ^11^ and imaging-based in situ platforms (e.g., Xenium, CosMx), ^12^ in terms of resolution, panel breadth, capture area, and typical use-cases, ^13^ distills pre-analytics and quality assurance (QC) steps aligned to histology workflows, ^14^ outlines ROI design, registration, segmentation, and analysis “recipes” that survive peer review, ^15^ and summarises validation strategies, including orthogonal assays and multi-site replication. ^16– 18^ By anchoring recommendations in platform documentation and recent translational reviews, the focus remains on choices that are feasible in FFPE and compatible with routine pathology. ^19^

**Figure 1.**
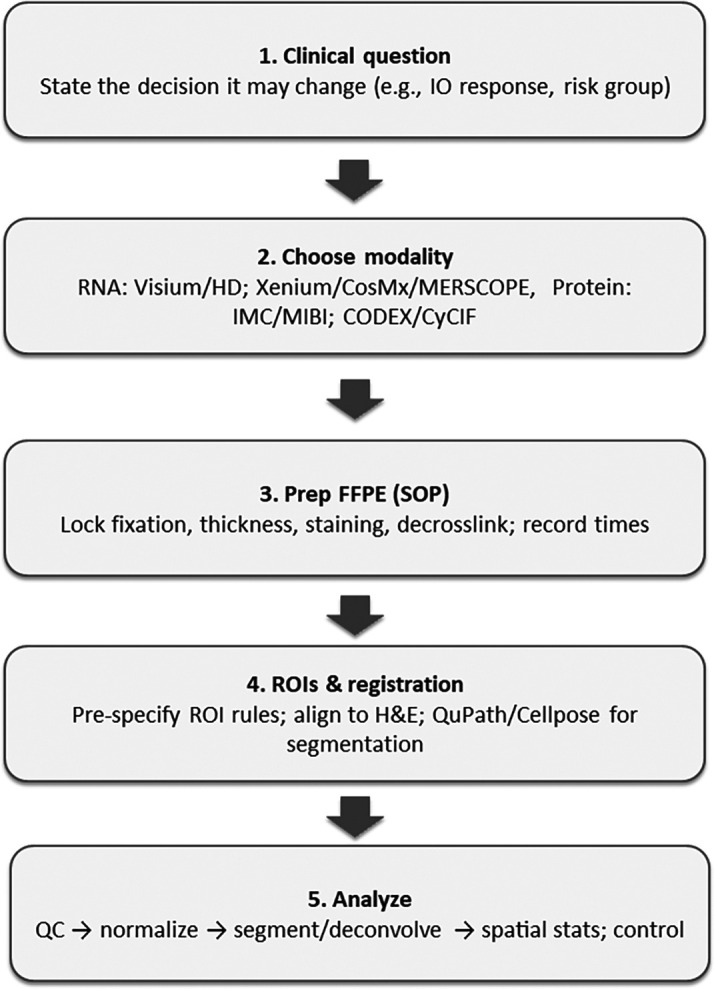
Spatial and haematoxylin and eosin analysis in formalin-fixed paraffin-embedded: streamlined vertical workflow from decision-making to reporting.

The aim of this mini-review is to provide a step-by-step guide for integrating spatial omics with routine H&E in FFPE specimens so teams can select a fit-for-purpose modality, implement robust pre-analytics and QC, plan analyses that generalize across sites, and structure validation and reporting to accelerate translational impact. Figure 1 summarises the end-to-end FFPE spatial workflow, which progresses from decision definition, modality selection, pre-analytical standardization, ROI and registration specification, analysis with QA gates, to validation and reporting. This sequence organizes the subsequent sections.

## Methodological approach

This mini-review was developed through an iterative and expert-guided process to synthesize the most relevant and current evidence. Our literature search was conducted up to September 01, 2025, primarily involving querying databases such as PubMed, Scopus, and Google Scholar. Key search terms included “spatial omics,” “FFPE,” “H&E,” “clinical pathology,” “workflow,” “reproducibility,” and “guidelines”. Our eligibility criteria for inclusion centered on direct relevance to integrating spatial omics with routine H&E in FFPE specimens, with an emphasis on translational and clinical applicability, methodological rigor, and actionable insights for pathology and research teams. Exclusion criteria included studies not utilizing FFPE samples, research focused solely on non-human models without clear translational links, purely theoretical or computational methods without experimental validation, and reviews lacking primary data synthesis or specific workflow recommendations. The evidence types included primary research articles, comprehensive reviews, manufacturer technical documents, and consensus guidelines, selected to ensure a balanced perspective on the current state and challenges of the field.

## Platforms for FFPE pathology: what actually works

Spatial assays available for deployment on archival FFPE tissue can be categorized into two broad classes. Sequencing-based spatial transcriptomics (ST), e.g., 10x Visium HD (FFPE), captures spot-based whole-transcriptome profiles registered to H&E, trading single-cell resolution for large capture areas and broad gene coverage. ^20^ In situ imaging platforms, e.g., 10x Xenium and NanoString CosMx SMI, measure targeted RNA (and, for CosMx, proteins) at single-cell or subcellular resolution on FFPE sections. ^21, 22^ MERFISH/MERSCOPE (Vizgen) is another high-plex in situ option with FFPE support. ^23^ For multiplex spatial proteomics, laboratories commonly use Imaging Mass Cytometry (IMC), Multiplexed Ion Beam Imaging (MIBI), ^24, 25^ or cyclic immunofluorescence systems such as Lunaphore COMET, MACSIMA, PhenoCycler Fusion (formerly CODEX) and CyCIF; ^26^^–^^28^ these often align naturally with IHC-centric diagnostic questions. Good platform overviews for pathologists are now available, alongside manufacturer FFPE handbooks. Key specifications are summarised in Table 1: sequencing-based “spot/grid” assays (e.g., 10x Visium FFPE/Visium HD) provide whole-transcriptome discovery over 6.5 × 6.5 mm capture areas (Visium 55 μm spots; HD 2 μm pixel output, typically binned), well suited to archival cohort screens and tumour–stroma mapping. ^29, 30^ In situ RNA imaging (10x Xenium, NanoString CosMx SMI, Vizgen MERSCOPE) yields targeted single-cell/subcellular maps (CosMx up to ~6,000 RNAs; MERSCOPE up to ~1,000) for pathway-focused profiling, immune-niche interrogation, and cross-validation with IHC/RNAscope. ^31– 33^ Multiplex spatial proteomics (IMC, MIBI, CODEX, CyCIF) complements RNA by quantifying proteins at single-cell resolution for immune phenotyping and actionable signatures. ^34^

**Table 1.** Formalin-fixed paraffin-embedded -compatible spatial omics platforms: a concise comparison for routine pathology.

Class	Representative platforms	Nominal resolution	Analyte	Panel breadth	Capture area/FOV	Typical throughput	Typical use-cases	References
Sequencing-based ST (spot/grid)	10x Visium FFPE/Visium HD	Visium: 55 μm spots; HD: 2 μm pixel output (binned for analysis)	RNA (whole-transcriptome)	Whole-transcriptome	Up to 4 capture areas/slide (~6.5 × 6.5 mm each)	Tens of sections per run (scanner + NGS dependent)	Discovery in archival cohorts; tumor–stroma programs; hypothesis generation	^29, 30^
In-situ RNA imaging	10x Xenium; NanoString CosMx SMI; Vizgen MERSCOPE	Single-cell/subcellular	RNA (targeted); CosMx also protein	Hundreds–thousands RNAs (CosMx up to ~6,000; MERSCOPE up to ~1,000); CosMx ~64–76 proteins	Tile-based FOVs; user-selected ROIs; multi-tile mosaics	~1–10 slides/week (instrument dependent)	Targeted pathway panels; immune niches; cross-validation with IHC/RNAscope	^31– 33^
Multiplex spatial proteomics	IMC; MIBI; CODEX; CyCIF	Single-cell	Protein (antibody panels)	~30–60+ markers (panelized)	Tile ROIs; mm ^2^–cm ^2^ mosaics	~1–10 slides/week	Immune phenotyping; actionable protein signatures; trial correlative studies	^34^

ST, spatial transcriptomics; FFPE, formalin-fixed paraffin-embedded; HD, high-definition pixel output (Visium HD); NGS, next-generation sequencing; RNA, ribonucleic acid; FOV, field of view; ROI, region of interest; IHC, immunohistochemistry; RNAscope, RNA in situ hybridization; SMI, Spatial Molecular Imager; IMC, imaging mass cytometry; MIBI, multiplexed ion beam imaging; CODEX, CO-Detection by indEXing; CyCIF, cyclic immunofluorescence; μm, micrometres; mm ^2^, square millimetres; cm ^2^, square centimetres.

## Pre-analytics & tissue handling: small decisions, big effects

FFPE spatial assays are highly sensitive to pre-analytics. ^35^ Follow platform-specific guidance on section thickness, deparaffinisation, H&E/IF staining, decrosslinking, and storage; these steps strongly influence RNA integrity, probe binding, and downstream quantification. ^36^ Common failure modes at this stage include significant RNA degradation due to improper handling or storage, high tissue autofluorescence, and compromised signal from necrotic or hemorrhagic regions, all of which necessitate careful pre-analytical assessment. ^37^ For example, the Visium HD FFPE handbook and Xenium FFPE guide detail slide prep, staining, and decrosslinking workflows ^29^; MERSCOPE provides FFPE-specific drying and storage advice. Critically, enzymatic steps can backfire: excess Proteinase-K in GeoMx DSP improved total reads but increased negative probe counts and reduced signal-to-noise, ultimately decreasing genes detected, highlighting why labs should pilot enzyme conditions and lock them before a study. ^33^ Such enzyme optimization is a key QC checkpoint to avoid compromising data quality.

## ROI selection, registration & segmentation

ROI strategy should be hypothesis-driven (e.g., tumour–stroma interfaces, immune niches, invasive fronts) and traceable back to H&E. ^38^ Platforms such as GeoMx and in situ imagers emphasize explicit ROI selection; document criteria prospectively. ^39^ Register spatial layers to H&E. Critical QC checkpoints during registration are needed to detect and correct for registration artifacts, which can lead to misaligned spatial data and erroneous interpretations. This step should be followed by validated, reproducible segmentation—QuPath remains a robust open-source WSI toolset for nuclei/cell detection, while Cellpose (and its newer variants) generalizes well across staining modalities with minimal tuning. ^40^ When publishing multiplex imaging data, adhere to the Minimum Information about Highly Multiplexed Tissue Imaging (MITI) standard so ROIs, acquisition parameters, and processing are transparent and reusable. ^41, 42^

## Analysis workflows that survive peer review

Successfully navigating complex and rapidly evolving analytical workflows requires significant multidisciplinary expertise, integrating pathology knowledge with dedicated advanced computational and statistical skills. Given the dynamic development of new tools, a cautious and critical approach is paramount. For spot-based spatial transcriptomics, robust analysis pipelines typically involve 1) quality control (QC) and normalization, ^43, 44^ to detect and mitigate issues like batch effects and low signal-to-noise, 2) deconvolution of spots using scRNA-seq references, ^45, 46^ and 3) testing for spatial associations. ^47^ Recent benchmarking studies across dozens of datasets consistently recommend methods such as cell2location, CARD, and Tangram for their high performance. ^48^ As new methods emerge, it is crucial to continuously evaluate and base choices on robust benchmarking evidence, recognizing that the optimal toolset can shift over time. Dedicated computational scientists or bioinformaticians with expertise in spatial data analysis are essential for proper tool selection, implementation, and interpretation. Multi-slice or multi-cohort integration benefits from modern alignment tools and requires careful reporting of cross-slide consistency to ensure robust findings. ^49^ For imaging proteomics, ensuring reproducible results necessitates rigorous denoising, batch correction, and neighbourhood analysis. ^50^ Recent best-practice guidelines in oncology provide comprehensive end-to-end pipelines, from acquisition and segmentation to phenotyping and spatial statistics. ^9, 48, 51, 52^

## Validation & reproducibility

Translational claims require orthogonal validation (e.g., RNAscope/IHC for RNA/protein hits), multi-site replication, and pre-registered analysis plans. ^53^ Use reporting checklists from pathology-facing reviews and adopt MITI for multiplex imaging so images, masks, and metadata are reusable. ^40^ Where possible, include an external test set (a different scanner/site or archival cohort) and quantify agreement (e.g., correlation of cell-type abundance, niche frequency). ^54^ High-level clinical perspectives emphasize linking spatial findings to outcomes or therapeutic response, not just discovery. ^55^

## Costs, throughput, and choosing RNA vs protein maps

For budgeting and platform choice, a comparative analysis of assay chemistry, resolution, capture area/fields per run, and instrument time rather than chasing absolute prices (which vary by site and service contract). As a guide, instrument cost can be considered high (>$500,000), medium ($100,000–$500,000), or low (<$100,000); per-sample cost high (>$1,000), medium ($100–$1,000), or low (<$100). ^56^ Protein-centric maps (multiplex IHC/IF) often deliver faster, lower per-slide costs for focused questions (e.g., immune phenotyping), ^57^ whereas whole-transcriptome ST (UMI-based RNA profiling) is better for unbiased discovery and retrospective cohorts. Resolution needs, spot vs single-cell/subcellular, and capture area (including the effective pixel/“bin,” e.g., 100 μm ^2^) determine run time and sequencing/imaging depth. ^58^ Above all, FFPE compatibility and workflow fit (embedding within existing histology/QC) should drive selection; LCM remains useful for targeted validation or rare regions. ^59^ Manufacturer documents (e.g., 10x Genomics, NanoString, Vizgen) summarize throughput, section prep, and run constraints that materially affect real-world cost and turnaround. ^12, 60, 61^

## Adoption roadmap for pathology services

Initial implementation should proceed incrementally. This involves defining a narrow clinical question and the specific decision it aims to influence, selecting a single FFPE-compatible platform, and standardizing pre-analytics. It is essential to formally establish ROI rules and lock down segmentation protocols, pre-register analyses, and plan orthogonal validation. Adherence to the MITI standard for data and metadata is crucial. Incorporating a multi-site or external test component as early as feasible is recommended. ^62, 63^ Recent best-practice frameworks in multiplex imaging/spatial biology, plus pathology-specific reviews, offer valuable checklists that can be adapted for institutional standard operating procedures (SOPs) and QA documents. ^24^

## Clinical exemplars

Below are brief, real-world examples illustrating the potential of pairing spatial omics with routine H&E to generate clinically relevant hypotheses and provide novel insights. By revealing precise molecular events within tissue architecture, these studies suggest how future validated applications could help clinicians refine biopsy approaches, improve risk stratification, and identify potential therapeutic targets, often revealing details missed by routine H&E or bulk assays. It is important to note that many of these applications are currently exploratory and require further rigorous validation to establish routine clinical utility and measurable endpoints.

In cutaneous squamous cell carcinoma, pairing spatial omics with H&E revealed where distinct tumour programmes live and whom they talk to. Integrated single-cell RNA-seq, spatial transcriptomics, and multiplexed ion-beam imaging mapped four tumour subpopulations, including a tumour-specific keratinocyte (TSK) state that localises to a fibrovascular niche on the H&E slide. Spatial mapping of ligand–receptor networks showed TSK cells act as a communication hub, while Tregs co-localized with CD8 T cells in compartmentalized stroma, an immunosuppressive arrangement you could miss with bulk profiling. Functionally, CRISPR screens flagged subpopulation-enriched networks as essential for tumourigenesis. Clinically, these H&E-anchored spatial readouts offer insights that could potentially guide biopsy targeting (e.g., sampling the TSK/fibrovascular interface), suggest refinements for risk stratification (e.g., based on presence/extent of Treg–CD8 niches), and nominate actionable pathways for exploratory trials focused on interrupting TSK-driven signalling or collapsing immunosuppressive neighbourhoods. ^64^

In pancreatic ductal adenocarcinoma, overlaying spatial proteomics on the H&E slide mapped the tumour microenvironment into 10 distinct neighbourhoods, including a vascular niche within PDAC’s characteristically hypovascular, hypoxic stroma. Across 35 H&E-guided ROIs from 9 patients (>140k cells, 26-marker imaging mass cytometry), the study localized where tumour proliferation concentrates and how immune subsets interface with vessels. Crucially, the vascular niche was tightly linked to CD44 ^+^ macrophages with a pro-angiogenic programme, nominating a microenvironmental target that standard bulk assays would miss. Clinically, these H&E-anchored spatial readouts provide valuable information that may help guide biopsy targeting (e.g., sampling vascular niches), contribute to sharper assessments of risk stratification (e.g., proliferative/immune-vascular interfaces), and inform future trial design for anti-angiogenic or macrophage-modulating combinations in PDAC. ^65^

In fatal COVID-19 lung disease, FFPE spatial transcriptomics (GeoMx) co-registered to H&E pinpointed patchy, non-uniform SARS-CoV-2 distribution and localised host responses to the exact anatomic foci. Areas with high viral load on the slide showed amplified type I interferon signaling, alongside broader upregulation of inflammation, coagulation, and angiogenesis pathways, patterns a bulk assay would blur. After controlling for dominant cell types and inter-patient variability, only a few genes distinguished COVID-19 from fatal influenza, but IFI27 remained significantly higher in COVID-19, reinforcing its value as a tissue-level biomarker that aligns with blood-based diagnostics. Clinically, these H&E-anchored spatial readouts provide valuable insights that could inform targeted sampling (e.g., of multiple foci rather than single cores), potentially support triage/therapy considerations by identifying interferon-rich, highly infected regions, and aid in the validation of biomarkers like IFI27 within diseased lung architecture. ^66^

In human dorsolateral prefrontal cortex, H&E-anchored spatial transcriptomics (10x Visium) mapped the six cortical layers and uncovered layer-enriched gene programmes, refining classic laminar markers on the same slide. Overlaying these maps onto single-nucleus RNA-seq re-grounded molecular clusters in real anatomy, improving interpretability. Clinically relevant gene sets for schizophrenia and autism showed layer-specific enrichment, offering clues to circuits and cell layers most implicated in disease. These insights suggest potential directions for targeted sampling, neuropathology reporting, and the design of hypothesis-driven trials (e.g., for layer-aware biomarkers or neuromodulation targets). Data-driven clustering, using H&E context, further assists in defining spatial domains in tissues with less obvious architecture. ^67^

In periodontitis, H&E-anchored spatial transcriptomics resolved gingival tissue into epithelium, inflamed connective tissue, and non-inflamed connective tissue on the same slide, revealing 92 genes upregulated specifically in inflamed zones. Top signals, IGLL5, SSR4, MZB1, XBP1, point to a B-cell/plasma-cell–rich, high-secretory programme and were validated by RT-qPCR and IHC. Clinically, these maps offer the potential for dentists and pathologists to target biopsies to truly active lesions, help differentiate active vs quiescent sites for risk stratification and follow-up, and enable tracking of response to therapy using compartment-specific markers, insights that bulk profiling often obscures. ^68^

In melanoma lymph node metastases, H&E-anchored spatial transcriptomics (10x Visium) sequenced >2,200 tissue domains and, after deconvolution, linked gene programmes to specific histological entities on the slide. This revealed coexisting melanoma transcriptional signatures within single regions and defined lymphoid niches adjacent to tumour with distinct expression patterns, heterogeneity not evident on morphology alone. Clinically, such maps can refine biopsy targeting (sample mixed-signature zones), sharpen staging/prognosis by quantifying tumour–immune interfaces, and inform immunotherapy strategies by identifying lymphoid areas most engaged with tumour. In short, pairing spatial omics with H&E reveals intratumoural and microenvironmental complexity that may suggest actionable avenues for research, details often missed by bulk profiling and routine histology. ^69^

In rheumatoid arthritis (RA) vs spondyloarthritis (SpA) synovium, H&E-anchored spatial transcriptomics let investigators zoom into mononuclear infiltrates on the slide and read out compartment-specific programmes. RA hotspots showed adaptive immune/T–B cell interaction signatures with enrichment of central memory T cells, whereas SpA regions favoured tissue-repair pathways with effector memory T cells. These H&E-guided spatial maps, supported by IHC and in silico cell-type calls, suggest practical avenues to refine biopsy targeting, aid in differential diagnosis when histology overlaps, and potentially inform therapy choices (e.g., B/T-cell-directed strategies in RA vs repair-oriented pathways in SpA) while enabling site-specific response monitoring. ^70^

In leprosy, pairing spatial omics with H&E turned granulomas from a uniform “mass” on the slide into an organised, layered architecture with distinct cellular and functional zones. By integrating single-cell and spatial sequencing on biopsies from reversal reactions (RRs) versus lepromatous disease (L-lep), the study localised interferon-γ/IL-1β–regulated antimicrobial programmes to specific niches where macrophages, T cells, keratinocytes, and fibroblasts cooperate. Clinically, H&E-anchored maps can provide valuable information to consider for targeted sampling of active antimicrobial layers during RR, inform future biomarker development for treatment monitoring (e.g., spatially resolved antimicrobial gene sets), and suggest pathways for therapy tailoring by highlighting sites most likely to respond to host-directed or immunomodulatory interventions, revealing granularity that bulk assays or morphology alone would typically miss. ^71^

In Amyotrophic Lateral Sclerosis (ALS) cortex, H&E-anchored spatial transcriptomics (∼100 μm spots) preserved laminar and regional anatomy on the slide, letting investigators pinpoint where disease programmes reside rather than averaging them out. Mapping post-mortem motor cortex from a C9orf72 case, then validating with BaseScope ISH and an extended cohort (sALS, SOD1, C9orf72), they found 16 dysregulated transcripts spanning six disease pathways and converged on two spatially dysregulated genes, GRM3 and USP47, consistently altered across ALS genotypes. Clinically, these H&E-registered maps contribute to explaining selective regional vulnerability, can inform the nomination of region-aware diagnostic markers and therapeutic targets, and may guide targeted sampling in neuropathology, revealing insights that bulk RNA or dissociated single-cell data would likely miss. ^72^

In another ALS, H&E-anchored spatial transcriptomics mapped the spinal cord’s molecular shifts across disease time in mice and in human post-mortem tissue, revealing when and where key pathways turn on. The maps distinguished regional microglia vs astrocyte programmes early in disease, and identified transcriptional pathways shared between murine models and human cords, signals that bulk RNA or dissociated cells would blur. Clinically, these slide-localized readouts can guide targeted sampling (vulnerable ventral horn regions), sharpen biomarker development (region- and cell-state markers for progression), and inform trial design/stratification (e.g., for enrolling patients by pathway-active niches), and can assist in aligning therapeutic timing with the actual spatial order of neuroinflammatory events. ^73^

## Limitations of spatial omics

The limitations of spatial omics often manifest as specific workflow failure modes that require careful attention and robust QC strategies to address. First, there are technology trade-offs on FFPE sections between map resolution, number of targets, and area covered ^74^: whole-transcriptome spot/grid methods lose single-cell precision, targeted in situ platforms measure fewer genes, and multiplex proteomics depends on well-validated antibodies. ^75^ Second, results are sensitive to pre-analytics, fixation quality, section thickness, and deparaffinisation/de-crosslinking, where too little or too much enzyme treatment degrades data; even storage time of cut slides matters. ^76– 78^ Certain tissues are difficult: decalcified bone often has fragmented RNA, necrotic/bleeding areas give weak signal, and highly pigmented/autofluorescent tissues (e.g., melanoma, lipofuscin-rich) can confound fluorescence without mitigation. ^67, 79^ Third, study design and sampling can introduce bias if ROI rules are not pre-specified and auditable (MITI) ^51, 80^; small ROIs may be underpowered and single-slide studies face slide/batch variability, so plan power, use multiple slides, and model batch. ^81– 83^ Retrospective cohorts may hide clinical/treatment confounders, so follow Strengthening the Reporting of Observational Studies in Epidemiology (STROBE)/Reporting Recommendations for Tumor Marker Prognostic Studies (REMARK) principles. ^84, 85^ Fourth, quantification and analysis have pitfalls: segmentation/cell calling remains error-prone and software/version changes can shift results, pipelines and parameters should be locked. ^86, 87^ For spot-based data, deconvolution depends on single-cell references that may not match tissue/platform/disease and can bias estimates ^88– 90^; batch effects (slide/run/site) can masquerade as biology without careful normalization/integration ^91^; testing thousands of features inflates false positives unless False Discovery Rate (FDR) is controlled and primary hypotheses are pre-registered. ^92^ Fifth, validation and generalizability are limited by variable cross-platform concordance (sequencing- vs imaging-based), so orthogonal confirmation (RNAscope/IHC) is important ^67, 93^; many studies stop at discovery rather than prospective, multi-site validation with outcomes and REMARK-aligned reporting. ^18, 94^ Sixth, critically, major operational and regulatory barriers currently prevent widespread routine clinical deployment, including substantial issues such as cost, compute/storage, and turnaround (e.g., Visium FFPE/HD depth; multi-TB images and QC) (52,95), ^52, 95^ significant site-to-site differences in infrastructure/training/QA that hinder reproducibility, ^96^ and the demanding and extensive need to move from Research Use Only/Laboratory-Developed Test (RUO/LDT) to IVD through Clinical Laboratory Improvement Amendments/College of American Pathologists (CLIA/CAP)-level validation with ongoing monitoring for assay/model drift. ^97^

## Future directions

For pathology laboratories, the most immediate and impactful path forward for spatial omics is to exploit the opportunity to use their huge archival resources to address important clinical questions on retrospective, well-curated cohorts of samples. This focus on high-quality translational research, rather than immediate IVD accreditation for routine clinical use (which remains unrealistic now, given cost and complexity), is crucial. To enable this, we need simple shared rules for data collection and reporting, using MITI-style metadata/checklists and multi-site harmonization, so ROI choices are auditable and datasets can be compared across studies. ^4, 67^ Crucially, this will require pathologists to work closely with technologists and data analysts/bioinformaticians to solve outstanding questions. We also need better ways to integrate platforms: align whole-transcriptome maps with targeted in situ RNA and multiplex proteomics on serial sections, and quantify uncertainty in those integrations, building on recent cross-technology benchmarks. ^94, 98, 99^ Clinical adoption for robust translational insights and eventual clinical utility will require prospective, multi-site studies with predefined endpoints, critically leveraging large archival FFPE resources and standardized retrospective cohorts with extensive cross-site replication, and reporting aligned to biomarker standards such as REMARK. ^100^ In this context, the primary focus for the near term remains on enabling high-quality translational research that meticulously addresses these prerequisites for future clinical translation and IVD readiness. End-to-end automation and QA, registration, segmentation/cell calling, deconvolution, batch correction, with version-locked code and continuous QC dashboards should be standard. ^43, 101^ Practical multi-omic co-detection protocols (RNA–protein now, metabolites later) on FFPE, paired with orthogonal validation (RNAscope/IHC), will increase confidence. ^87, 102^ Finally, improving cost and throughput, through batching, smart ROI strategies, and targeted panels, will be essential to meet clinical turnaround times and facilitate the extensive validation required for clinical translation, prior to any consideration of IVD certification. Recent work outlines feasible high-throughput paths. ^40, 42^

## Conclusions

Spatial omics now complements routine H&E on FFPE tissue and can answer clinically relevant questions about tumour–immune architecture, heterogeneity, and microenvironmental niches. Effective adoption for translational research and eventual clinical use hinges on four critical elements emphasized in this mini-review: (1) fit-for-purpose platform selection (RNA vs protein; discovery vs targeted), (2) disciplined pre-analytics and QC, (3) transparent ROI, registration, and analysis workflows that are locked and auditable, and (4) robust orthogonal validation and multi-site replication to support rigorous translational claims. Framing studies around decisions that matter to clinicians (diagnosis, risk stratification, therapy selection) will ultimately accelerate real-world impact once these foundational challenges are comprehensively addressed.

## Data Availability

There are no underlying data associated with this article.
