# Supplementary material for: Integrating spatial omics with routine haematoxylin and eosin in formalin-fixed paraffin-embedded: a step-by-step clinical workflow
Source: F1000Res. 2026 Jan 19;14:1057. Originally published 2025 Oct 9. [Version 2] doi: 10.12688/f1000research.170680.2 (PMC12881848; doi:10.12688/f1000research.170680.2)
Supplement: Peer review history

# Reviewer report: Reviewer response for version 2

**DOI:** 10.5256/f1000research.195156.r452525

**Published:** 2026-02-06

**Authors:** Xu Xun

**Affiliations:** 1 State Key Laboratory of Genome and Multi-omics Technologies, BGI Research, Shenzhen, China

General Assessment:

 This mini-review addresses a timely and critical topic. However, specifically for Version 2, the manuscript requires sharper delineation between "research utility" and "clinical readiness," and needs to provide more concrete guidance on the technical bottlenecks of FFPE implementation.

 Major Comments

 1.    Sharpen clinical positioning and recalibrate expectations:

 The clinical exemplars currently blur the line between hypothesis-generating research and actionable clinical insight. Phrases like "guide biopsy targeting" imply near-term utility that is currently unsupported by cost/benefit or regulatory evidence. The authors must explicitly label the research phase of these technologies (e.g., "Exploratory" vs. "Validation") and add disclaimers regarding the gap between biological relevance and clinical readiness.

 2.    Deepen methodological transparency regarding selection bias: 

 While the new "Methodological Approach" section is noted, it lacks critical analysis regarding selection and publication bias. The literature heavily favors successful experiments on high-quality samples. The review must acknowledge that "step-by-step workflows" derived from these papers often overlook the high failure rates encountered with real-world archival blocks (e.g., block age >5 years, variable fixation). Please discuss these limitations to avoid survivorship bias in your recommendations.

 3.    Guide cross-platform decisions with a decision matrix:

 Synthesize the benchmarking literature to characterize batch effects and expand Figure 1 into a Decision Matrix. This should match the modality not just to the research question, but to sample constraints (e.g., "If RNA integrity is low, prioritize Modality X over Modality Y").

 4.    Distinguish H&E workflows: Same-section vs. Adjacent-section:

 This is a critical technical distinction often overlooked. Same-section (e.g., Visium) versus adjacent-section (e.g., many proteomic panels) approaches carry distinct analytical consequences for registration error tolerance and segmentation parameterization. These distinctions must be articulated explicitly, accompanied by empirically grounded quality control thresholds for registration accuracy.

 5.    Stratify operational feasibility beyond technical specs: 

 Instead of a theoretical framework, please stratify the clinical applicability based on Operational Readiness. Analyze the specific translational bottlenecks for each technology class, such as Turnaround Time (TAT) pressures, computational infrastructure requirements (e.g., local server vs. cloud compliance), and the complexity of CLIA-level validation.

 6.    Elevate H&E as an independent modality:

 The manuscript currently treats H&E predominantly as a spatial anchor. It is recommended that the "Future Directions" section explicitly state the value of integrating quantitative morphometric features (Computational Pathology) with molecular data, rather than viewing H&E merely as a background map.

 Minor Comments

 1.    Pre-analytical QC Metrics: Please specify concrete quality control metrics for FFPE tissues. For instance, discuss the role of DV200 scores in determining sample eligibility for transcriptomic assays, rather than just generic "fixation quality."

 2.    Nomenclature Consistency: Ensure platform names are current and consistent (e.g., clarify "CODEX" vs. "PhenoCycler Fusion" usage throughout).

 3.    Table 1 Coverage: In Table 1, please clarify the "effective coverage" or "gap area" for spot-based transcriptomics, as this is a material limitation for detecting rare niches compared to imaging-based methods.

Is the review written in accessible language?

Yes

Are all factual statements correct and adequately supported by citations?

Yes

Are the conclusions drawn appropriate in the context of the current research literature?

Partly

Is the topic of the review discussed comprehensively in the context of the current literature?

Partly

Reviewer Expertise:

my area of expertise involves the development of core instrumentation and technologies for DNA sequencing and synthesis, with a specific focus on single-cell sequencing and spatial omics technologies. Additionally, I am dedicated to the translational application of these advanced methods across diverse fields, including synthetic biology, clinical disease diagnosis and treatment.

I confirm that I have read this submission and believe that I have an appropriate level of expertise to confirm that it is of an acceptable scientific standard, however I have significant reservations, as outlined above.

# Reviewer report: Reviewer response for version 2

**DOI:** 10.5256/f1000research.195156.r451194

**Published:** 2026-01-28

**Authors:** Marie-Liesse Asselin-Labat

**Affiliations:** 1 Walter and Eliza Hall Institute of Medical Research, Melbourne, Australia

The author has addressed my previous queries. I have no further comments.

Is the review written in accessible language?

Yes

Are all factual statements correct and adequately supported by citations?

Yes

Are the conclusions drawn appropriate in the context of the current research literature?

Partly

Is the topic of the review discussed comprehensively in the context of the current literature?

Partly

Reviewer Expertise:

cancer biology, spatial omics

I confirm that I have read this submission and believe that I have an appropriate level of expertise to confirm that it is of an acceptable scientific standard.

# Reviewer report: Reviewer response for version 2

**DOI:** 10.5256/f1000research.195156.r451193

**Published:** 2026-01-20

**Authors:** Da-Wei Yang

**Affiliations:** 1 Fudan University, Shanghai, China

The authors have addressed all comments point by point, and I recommend acceptance of the current version.

Is the review written in accessible language?

Yes

Are all factual statements correct and adequately supported by citations?

Yes

Are the conclusions drawn appropriate in the context of the current research literature?

Yes

Is the topic of the review discussed comprehensively in the context of the current literature?

Yes

Reviewer Expertise:

Early lung cancer, pulmonary nodules, LDCT screening, thoracic imaging AI, radiomics, clinical decision support, biomarkers, multi-omics, liquid biopsy, NK cells, tumor microenvironment, single-cell RNA-seq, spatial transcriptomics, digital health, IoT respiratory medicine, medical simulation/metaverse medicine.

I confirm that I have read this submission and believe that I have an appropriate level of expertise to confirm that it is of an acceptable scientific standard.

# Reviewer report: Reviewer response for version 1

**DOI:** 10.5256/f1000research.188170.r438425

**Published:** 2025-12-31

**Authors:** Da-Wei Yang

**Affiliations:** 1 Fudan University, Shanghai, China

This FFPE-focused mini-review provides a pragmatic, clinic-facing roadmap for integrating spatial omics with routine histopathology, organized around a decision-first workflow (define the clinical decision → select modality → lock pre-analytics → pre-specify ROIs/registration → analyze with QA gates → validate/report). The manuscript appropriately emphasizes pre-analytical sensitivity, ROI strategy, and the importance of reporting standards (e.g., MITI; STROBE/REMARK). However, the current framing overstates near-term readiness for routine pathology/IVD deployment, and the review would benefit from (i) more cautious clinical positioning, (ii) a minimal, transparent review methodology, (iii) updated platform coverage and nomenclature, and (iv) more benchmark-anchored guidance in the analytics section.

**Recommendation: Major revision.**

 MAJOR COMMENTS

1. Clinical positioning should be recalibrated: statements implying near-term compatibility with routine pathology/IVD deployment are overly optimistic given cost, operational complexity, cross-site reproducibility, and regulatory considerations.
2. The review lacks minimal methodological transparency: a brief description of search strategy, eligibility criteria, and evidence typing is needed to support claims of coverage and reduce concerns about selection bias.
3. Platform landscape coverage and naming require updating: spatial proteomics platforms should be more complete and nomenclature should be corrected/standardized (e.g., Phenocycler Fusion, formerly CODEX).
4. The analytics section needs tightening and stronger evidentiary grounding: remove drafting artifacts/second-person phrasing, temper prescriptive statements, and anchor recommendations to benchmarking/comparative evidence; emphasize reproducibility practices and multidisciplinary expertise.
5. Clinical exemplars should be more explicitly decision-linked: examples are informative but often remain hypothesis-generating; claims of clinical enablement should be supported by clearer decision points, validation pathways, and measurable endpoints, with explicit separation of established utility vs exploratory insights.
6. The Introduction should acknowledge IHC as central to current clinical decision-making and more clearly articulate the incremental value of spatial omics beyond H&E+IHC (e.g., high-plex co-localization, niches, gradients, architecture, objective quantification).
7. Future Directions should prioritize realistic near-term impact: leveraging large archival FFPE resources and standardized retrospective cohorts with cross-site replication, rather than implying near-term IVD certification.

MINOR COMMENTS

1. Define all abbreviations at first mention (e.g., ALS) and ensure consistency across text, figures, and tables.
2. Ensure consistent platform naming across the manuscript, including tables and figure legends.
3. Add brief workflow “failure modes and QC checkpoints” where relevant (e.g., autofluorescence, necrosis/hemorrhage, RNA quality variability, registration artifacts).
4. Perform a language pass to remove residual drafting artifacts and standardize tone to formal scientific narration.

Is the review written in accessible language?

Yes

Are all factual statements correct and adequately supported by citations?

Yes

Are the conclusions drawn appropriate in the context of the current research literature?

Yes

Is the topic of the review discussed comprehensively in the context of the current literature?

Yes

Reviewer Expertise:

Early lung cancer, pulmonary nodules, LDCT screening, thoracic imaging AI, radiomics, clinical decision support, biomarkers, multi-omics, liquid biopsy, NK cells, tumor microenvironment, single-cell RNA-seq, spatial transcriptomics, digital health, IoT respiratory medicine, medical simulation/metaverse medicine.

I confirm that I have read this submission and believe that I have an appropriate level of expertise to confirm that it is of an acceptable scientific standard, however I have significant reservations, as outlined above.

# Response

**Published:** 2026-01-12

**Authors:** Nasar Alwahaibi

**Affiliations:** Biomedical Science, Sultan Qaboos University, Muscat, Oman

**We would like to take this opportunity to express our thanks to the reviewer for the positive feedback and helpful comments.**

**Below are our responses, point-by-point to the queries of the reviewer.**

**Reviewer 2**

 This FFPE-focused mini-review provides a pragmatic, clinic-facing roadmap for integrating spatial omics with routine histopathology, organized around a decision-first workflow (define the clinical decision → select modality → lock pre-analytics → pre-specify ROIs/registration → analyze with QA gates → validate/report). The manuscript appropriately emphasizes pre-analytical sensitivity, ROI strategy, and the importance of reporting standards (e.g., MITI; STROBE/REMARK). However, the current framing overstates near-term readiness for routine pathology/IVD deployment, and the review would benefit from (i) more cautious clinical positioning, (ii) a minimal, transparent review methodology, (iii) updated platform coverage and nomenclature, and (iv) more benchmark-anchored guidance in the analytics section.

**Recommendation: Major revision.**

 MAJOR COMMENTS

1. Clinical positioning should be recalibrated: statements implying near-term compatibility with routine pathology/IVD deployment are overly optimistic given cost, operational complexity, cross-site reproducibility, and regulatory considerations.

**Response**

**We appreciate the reviewer's feedback regarding the clinical positioning of spatial omics. We fully agree that statements implying near-term, widespread compatibility with routine pathology or IVD deployment can be overly optimistic and must be carefully qualified. Our intention with this mini-review is not to suggest immediate readiness for routine clinical integration, but rather to provide a pragmatic, step-by-step roadmap for translational teams and pathology services to rigorously explore and validate spatial omics, guiding efforts toward its eventual clinical utility.**

**As suggested, we have recalibrated the clinical positioning throughout the text, incorporating changes as suggested into the abstract, introduction, limitations, future directions, and conclusion sections.**

  

1. The review lacks minimal methodological transparency: a brief description of search strategy, eligibility criteria, and evidence typing is needed to support claims of coverage and reduce concerns about selection bias.

**Response**

**As suggested, a new subsection, Methodological Approach, has been added.**

  

1. Platform landscape coverage and naming require updating: spatial proteomics platforms should be more complete and nomenclature should be corrected/standardized (e.g., Phenocycler Fusion, formerly CODEX).

Response

**As suggested, we have updated spatial proteomics platform nomenclature throughout the text, specifically corrected "CODEX" to "PhenoCycler Fusion," and ensure comprehensive coverage in the relevant sections and Table 1.**

  

1. The analytics section needs tightening and stronger evidentiary grounding: remove drafting artifacts/second-person phrasing, temper prescriptive statements, and anchor recommendations to benchmarking/comparative evidence; emphasize reproducibility practices and multidisciplinary expertise.

**Response**

**As suggested, the analytics section has been revised to remove drafting artifacts and second-person phrasing, temper prescriptive statements with evidentiary grounding, and emphasize reproducibility and multidisciplinary expertise.**

  

1. Clinical exemplars should be more explicitly decision-linked: examples are informative but often remain hypothesis-generating; claims of clinical enablement should be supported by clearer decision points, validation pathways, and measurable endpoints, with explicit separation of established utility vs exploratory insights.

**Response**

**Thank you for your comment, all clinical exemplars have been revised to be more explicitly decision-linked, clarify validation pathways, and separate established utility from exploratory insights as suggested.**

  

1. The Introduction should acknowledge IHC as central to current clinical decision-making and more clearly articulate the incremental value of spatial omics beyond H&E+IHC (e.g., high-plex co-localization, niches, gradients, architecture, objective quantification).

**Response**

**As suggested, the introduction section has been revised to acknowledge IHC's central role and explicitly articulate the incremental value of spatial omics beyond H&E+IHC.**

  

1. Future Directions should prioritize realistic near-term impact: leveraging large archival FFPE resources and standardized retrospective cohorts with cross-site replication, rather than implying near-term IVD certification.

**Response**

**The future directions section has been revised to prioritize leveraging archival FFPE resources and cross-site replication for near-term impact, distinguishing these steps from later IVD certification, as suggested.**

 MINOR COMMENTS

1. Define all abbreviations at first mention (e.g., ALS) and ensure consistency across text, figures, and tables.

**Response**

**The entire manuscript, including text, figure, and table, has been reviewed to ensure all abbreviations are defined at first mention and consistently used thereafter, as suggested.**

  

1. Ensure consistent platform naming across the manuscript, including tables and figure legends.

**Response**

**As suggested, consistent platform naming, including updates like PhenoCycler Fusion, has been ensured across the entire manuscript, including table and figure legends.**

  

1. Add brief workflow “failure modes and QC checkpoints” where relevant (e.g., autofluorescence, necrosis/hemorrhage, RNA quality variability, registration artifacts).

**Response**

**As suggested, Workflow "failure modes and QC checkpoints" have been incorporated into relevant sections, particularly within pre-analytics, ROI selection, and analysis, , to enhance the practical utility of the guide.**

  

1. Perform a language pass to remove residual drafting artifacts and standardize tone to formal scientific narration.

**Response**

**As suggested, the comprehensive language pass has been performed throughout the entire manuscript.**

**Thank you.**

# Reviewer report: Reviewer response for version 1

**DOI:** 10.5256/f1000research.188170.r427396

**Published:** 2025-11-20

**Authors:** Marie-Liesse Asselin-Labat

**Affiliations:** 1 Walter and Eliza Hall Institute of Medical Research, Melbourne, Australia

This review article provides an overview of spatial omics technologies and their potential use in pathology departments.

 In the introduction, the author indicates the transition from routine HandE for clinical decision making to spatial omics. The author should also acknowledge that IHC is used for many clinical decisions.

 It seems a bit unrealistic to think that spatial omics is poised to be compatible with routine pathology. Cost and complexity of the methodology and analyses are a major barrier for clinical uptake, far from cross-centre validation for clinical testing. The author should temper the introduction and the future direction to acknowledge that spatial omics is not ready for IVD and routine clinical application. The focus of the review may be more on enabling high-quality translational research than bringing spatial omics technologies to IVD and routine pathology.

 Lunaphore COMET and MACSIMA should be included in the spatial proteomic platforms with CODEX. CODEX has been renamed Phenocycler Fusion.

 There is a comment on page 4, in Analysis workflow stating: ‘your review should point readers to benchmark-grounded choices’. This sentence needs to be edited, and benchmarked tools provided.

 Analysis workflows are still very complex, and there are a number of new analytical tools being generated. This section should be written with caution to highlight the evolving analytical tools. Also, experts in these analysis methodologies should be involved in the analysis.

 The examples cited are interesting and highlight ongoing translational research.

 The future direction section may focus on the opportunity for pathology labs to exploit spatial omics technologies to use their huge archival resources to address important clinical questions on retrospective, well-curated cohorts of samples, rather than IVD accreditation for routine clinical use, which is unrealistic now, given cost and complexity. Pathology labs should use this opportunity to work with technologists and data analysts/bioinformaticians to solve outstanding questions.

 Please define ALS.

Is the review written in accessible language?

Yes

Are all factual statements correct and adequately supported by citations?

Yes

Are the conclusions drawn appropriate in the context of the current research literature?

Partly

Is the topic of the review discussed comprehensively in the context of the current literature?

Partly

Reviewer Expertise:

cancer biology, spatial omics

I confirm that I have read this submission and believe that I have an appropriate level of expertise to confirm that it is of an acceptable scientific standard, however I have significant reservations, as outlined above.

# Response

**Published:** 2026-01-12

**Authors:** Nasar Alwahaibi

**Affiliations:** Biomedical Science, Sultan Qaboos University, Muscat, Oman

We would like to take this opportunity to express our thanks to the reviewers for the positive feedback and helpful comments.

 Below are our responses, point-by-point to the queries of the reviewers.

**Reviewer 1**

 This review article provides an overview of spatial omics technologies and their potential use in pathology departments. In the introduction, the author indicates the transition from routine H and E for clinical decision making to spatial omics. The author should also acknowledge that IHC is used for many clinical decisions.

**Response:**

**As suggested, the introduction section has been revised to acknowledge IHC's central role for many clinical decisions.**

 It seems a bit unrealistic to think that spatial omics is poised to be compatible with routine pathology. Cost and complexity of the methodology and analyses are a major barrier for clinical uptake, far from cross-centre validation for clinical testing. The author should temper the introduction and the future direction to acknowledge that spatial omics is not ready for IVD and routine clinical application. The focus of the review may be more on enabling high-quality translational research than bringing spatial omics technologies to IVD and routine pathology.

**Response:**

**We appreciate your feedback on the readiness of spatial omics for routine clinical pathology. We fully agree that significant barriers (cost, complexity, validation, regulation) mean spatial omics is not yet ready for IVD or widespread clinical application.**

**Our review's primary focus is on providing a roadmap for high-quality translational research, which is a critical prerequisite for eventual clinical impact, not immediate clinical adoption.**

**We have refined the Introduction and Abstract sections to temper the overall tone and emphasize this translational research focus. Our Limitations' and Future Directions sections further elaborate on these crucial hurdles and the disciplined steps required for future clinical utility.**

 Lunaphore COMET and MACSIMA should be included in the spatial proteomic platforms with CODEX. CODEX has been renamed Phenocycler Fusion.

**Response:**

**Thank you for pointing out the omission of Lunaphore COMET and MACSIMA, and for the update regarding CODEX's renaming. We agree that these platforms are important to include for a comprehensive overview of spatial proteomics. We have updated the text in the 'Platforms for FFPE pathology: what actually works' section and Table 1 to reflect these additions and the correct nomenclature, specifically noting PhenoCycler Fusion (formerly CODEX).**

 There is a comment on page 4, in Analysis workflow stating: ‘your review should point readers to benchmark-grounded choices’. This sentence needs to be edited, and benchmarked tools provided.

**Response:**

**Thank you for your suggestion. We have incorporated it as requested.**

**On page 4, under 'Analysis workflows that survive peer review,' we now highlight benchmark-grounded tools: Recent benchmarking studies across dozens of datasets consistently recommend methods such as cell2location, CARD, and Tangram for their high performance (48).**

 Analysis workflows are still very complex, and there are a number of new analytical tools being generated. This section should be written with caution to highlight the evolving analytical tools. Also, experts in these analysis methodologies should be involved in the analysis.

**Response:**

**We agree that spatial omics analysis workflows are complex and rapidly evolving, requiring caution and expert involvement.**

**We've revised the 'Analysis workflows that survive peer review' section to emphasize this dynamic landscape, the continuous evaluation of tools, and the critical need for dedicated computational and statistical expertise.**

 The examples cited are interesting and highlight ongoing translational research.

**Response:**

**Thank you for this positive feedback.**

 The future direction section may focus on the opportunity for pathology labs to exploit spatial omics technologies to use their huge archival resources to address important clinical questions on retrospective, well-curated cohorts of samples, rather than IVD accreditation for routine clinical use, which is unrealistic now, given cost and complexity. Pathology labs should use this opportunity to work with technologists and data analysts/bioinformaticians to solve outstanding questions.

**Response:**

**Thank you for this highly relevant feedback. We fully agree that the immediate opportunity for pathology labs lies in leveraging archival resources for high-quality translational research, rather than immediate IVD accreditation, given current costs and complexity.**

**We have revised the Future directions section to explicitly emphasize this approach and highlight the critical need for collaboration between pathologists, technologists, and data scientists.**

**Thank you.**
